# Bowel Perforation in Premature Infants with Necrotizing Enterocolitis: Risk Factors and Outcomes

**DOI:** 10.1155/2016/6134187

**Published:** 2016-06-08

**Authors:** Lingling Yu, Jianmei Tian, Xingli Zhao, Ping Cheng, Xiaoqian Chen, Yun Yu, Xiaochun Ding, Xueping Zhu, Zhihui Xiao

**Affiliations:** ^1^Department of Neonatology, Children's Hospital of Soochow University, Suzhou 215003, China; ^2^Department of Infectious Disease, Children's Hospital of Soochow University, Suzhou 215003, China

## Abstract

We aim to determine risk factors and clinical outcomes for bowel perforation in premature infants with NEC. We analyzed clinical data of 57 cases of premature infants with NEC at our NICU between January 2010 and December 2012. Based on the presence of bowel perforation, we divided these infants into two groups: perforated NEC group (*n* = 10) and nonperforated NEC group (*n* = 47). We compared general information, clinical characteristics, and laboratory findings between groups. The perforated NEC group, compared to the nonperforated NEC group, had significantly lesser gestational age, lower birth weight, higher prevalence of apnea, mechanical ventilation, sepsis and shock, lower blood pH, higher levels of blood glucose, abnormal WBC count and thrombocytopenia, and elevated CRP (all *P* < 0.05). Moreover, the perforated NEC group had significantly longer durations of fasting and TPN usage, higher incidences of EUGR and cholestasis, longer duration of antibiotics, higher frequency of advanced antibiotics use, and poorer prognosis than the nonperforated NEC group (all *P* < 0.05). Bowel perforation in premature infants with NEC was associated with multiple risk factors. Early identification of some of these risk factors in premature infants with NEC may help implement early intervention to reduce the incidence of bowel perforation and thereby improve the prognosis.

## 1. Introduction

Necrotizing enterocolitis (NEC) is one of the devastating diseases in premature neonates [1–4]. A high incidence of NEC (7%) and related mortality (20–30%) has been reported in very low birth weight (VLBW) infants [1], and, in our hospital, the incidence of NEC was 5.54% [5]. Bowel perforation is a life-threatening complication of NEC in premature infants [6]. This complication is associated with mortality as high as 76% [7] and long-term complications among survivors, such as short bowel syndrome, growth and developmental retardation, and adverse neurological outcome [8].

Linder et al. [9] have found bowel perforation in NEC associated with postnatal age, abdominal distension, metabolic acidosis, higher blood glucose, and elevated liver enzymes. In extremely LBW infants, Wu et al. [10] found that bowel perforation was associated with thrombocytopenia, elevated C-reactive protein (CRP), and anemia. However, risk factors for bowel perforation in neonates with NEC are not well characterized.

In the current study, we aimed to determine potential risk factors and clinical outcomes of bowel perforation in premature infants with NEC.

## 2. Methods

### 2.1. Study Population

We used Bell's staging criteria [11] to define NEC stages: stages IA and IB for suspect NEC, stage II for definite NEC, and stage III for advanced NEC. We identified 57 cases of NEC (stage II and above) in premature infants admitted to the neonatal intensive care unit of Children's Hospital of Soochow University (Suzhou, China) from January 2010 to December 2012. We excluded 46 cases with incomplete clinical information and those with known digestive tract malformation. The Institutional Review Board of the Children's Hospital approved the study. Informed consent was obtained from the parents.

The diagnosis of bowel perforation in NEC cases was based upon clinical manifestation in combination with findings on dynamic abdominal X-ray studies and at surgery, such as pneumoperitoneum [12]. In seven cases, perforation was diagnosed by X-ray and in 3 cases perforation was identified at surgery. Based on the presence of bowel perforation, we divided the cases into two groups: perforated NEC group (*n* = 10) and nonperforated NEC group (*n* = 47). The treatment of NEC included fasting, gastrointestinal decompression, third-generation cephalosporins, and total parenteral nutrition (TPN).

### 2.2. Clinical and Laboratory Examinations

We collected data on demographical information, clinical characteristics, perinatal factors, and complications. Data included gender, gestational age, birth weight, 5-minute Apgar score, maternal diseases and conditions during pregnancy (pregnancy-induced hypertension or diabetes, premature rupture of membranes, placenta previa, and placental abruption), fetal distress, delivery mode, singleton or twin pregnancy, age of onset of NEC (days), time for the first breastfeeding, rapidity of increase of milk intake, neonatal respiratory distress syndrome (RDS), neonatal pneumonia, congenital heart disease (CHD), sepsis (early-onset sepsis defined as less than postnatal 72 hours; late-onset sepsis defined as later than postnatal 72 hours), apnea, intracranial hemorrhage (ICH), shock (infectious shock), blood transfusions, and mechanical ventilation. We also collected data on clinical symptoms including abdominal distension, vomiting, and bloody stools and extrauterine growth retardation (EUGR) at discharge (EUGR defined as the weight calculated on the basis of the correct gestational age less than their peers below the tenth percentile at discharge). Laboratory data included white blood cell count (WBC, abnormality defined as <5.0 or >20 × 10^9^/L), platelet count (thrombocytopenia defined as <100 × 10^9^/L), CRP (abnormality defined as >8 mg/L), arterial blood gas parameters, and blood glucose levels (hyperglycemia defined as blood glucose > 7.0 mmol/L).

Score for Neonatal Acute Physiology-II (SNAP-II) and SNAP-Perinatal Extension-II (SNAPPE-II) scoring systems [13] were estimated for all NEC cases within 24 hours of life. The simplified version of the 2001 SNAP-II scoring system [13] included six objective measures, namely, average blood pressure, the lowest body temperature, PO_2_/FiO_2_ ratio (oxygenation index), multiple twitches, urine volume, and the lowest serum pH. These measures plus birth body mass, Apgar score, and small for gestational age (SGA) comprised the SNAPPE-II scores.

### 2.3. Statistical Analysis

Data were analyzed by using SPSS v20 statistical software package (SPSS, Inc., Chicago, IL, USA). Normally distributed continuous data were expressed as mean ± standard deviation (SD), and differences between groups were tested by *t*-test. Continuous data which were not normally distributed were expressed as median and interquartile range and tested by using rank test for differences between groups. Numerical data were expressed by number and percentage within groups and tested by using the chi-square test. When theoretical frequency was <1 in the contingency table, Fisher's exact probability method was used, and when theoretical frequency was ≥1 and <5, continuity correction was carried out. A *P* value <0.05 was considered as statistically significant.

## 3. Results

Table 1 shows general characteristics of NEC infants with and without bowel perforation. Among the total 57 cases, 18 were male and 39 were female. Among the NEC group with perforation, 4 cases gave up treatment (all the 4 cases died according to our follow-up by telephone call), while in the NEC group without perforation, 9 cases gave up treatment due to economic reasons (6 died and 3 survived) (at the time of the decision to withdraw support, the infants were moribund and the prognosis was futile). The perforated NEC group (*n* = 10), compared to the nonperforated NEC group (*n* = 47), had a significantly lesser gestational age and lower birth weight (all *P* < 0.05). There were no significant differences regarding the distribution of gender, SGA, twin pregnancy, Apgar score, fetal distress, cesarean section, or maternal diseases between two groups (*P* > 0.05). In addition, we did not observe significant differences with respect to SNAP-II and SNAPPE-II scores between groups.

The perforated NEC group had significantly higher prevalence of apnea, sepsis, and shock and a more frequent utilization of mechanical ventilation than nonperforated NEC group (Table 2). There was one case of early-onset sepsis and 4 cases of late-onset sepsis in the perforated NEC group and there was no case of early-onset sepsis and 4 cases of late-onset sepsis in the nonperforated NEC group. Shock was found in 3 cases of perforated NEC group and in one case of nonperforated NEC group. Concomitance of sepsis and shock was found among 3 cases in the perforated NEC group and one case in the nonperforated NEC group. NEC onset age, time for the first enteral feeding, speed of increment of milk intake, the distribution of primary diseases, RDS, neonatal pneumonia, abdominal distension, vomiting, bloody stools, CHD, blood transfusion, and ICH were not significantly different between groups (*P* > 0.05).

With regard to laboratory tests (Table 3), all the data were obtained during the course of NEC and before the perforation; the perforated NEC group had a lower arterial blood pH and higher blood glucose levels than nonperforated NEC group. Meanwhile, the likelihood of abnormal WBC count, thrombocytopenia, and elevated CRP was significantly higher in the perforated NEC group compared to nonperforated NEC group.

Among the NEC perforated group, 2 cases died, 4 gave up treatment, and the remaining 4 cases were treated by surgery. The perforated NEC group, compared to the nonperforated NEC group, had significantly longer durations of fasting, TPN days, and antibiotics, higher incidences of extrauterine growth restriction (EUGR) and cholestasis, and higher frequency of antibiotics upgrade (Table 4).

**Figure 1:**
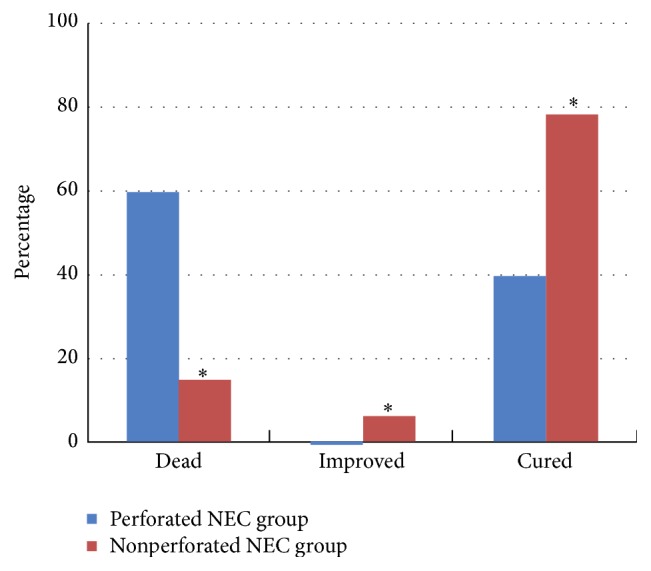
Clinical outcomes of NEC infants with and without bowel perforation. ^*∗*^ *P* < 0.05 compared to the nonperforated NEC group. “Cured” means complete functional recovery to a healthy state, while “Improved” means partial functional recovery with symptoms ameliorated, while some organ dysfunctions may remain.

During the clinical follow-up, all 4 cases in the perforated NEC group who gave up treatment died, and 2 of the 4 cases who underwent surgery had growth and development retardation. In the nonperforated NEC group, 1 case died, 9 gave up treatment (6 died and 3 survived by the telephone follow-up), and 8 of the other 37 cured cases showed growth and development retardation. The cure rate and mortality between the groups during follow-up were significantly (*P* < 0.05) different (Figure 1).

## 4. Discussion

In this study, we found that bowel perforation in premature infants with NEC was associated with multiple risk factors, such as lesser gestational age, lower birth weight, apnea, and sepsis. In addition, the perforated NEC group had higher prevalence of abnormal WBC count, thrombocytopenia, elevated CRP, and blood glucose levels than nonperforated NEC group. The bowel perforation was also associated with unfavorable prognosis. Previously, earlier postnatal age at NEC occurrence and lower birth weight were identified as high-risk factors for bowel perforation in NEC by Linder et al. [9].

NEC, especially complicated with bowel perforation, is the leading cause of deaths in preterm neonates [8]. Some studies have suggested a rapid increase of milk intake, asphyxia, maternal diseases, CHD, and blood transfusion as high-risk factors for NEC in premature neonates [14, 15]. However, it is unclear whether these factors influence bowel perforation in infants with NEC. We found no significant differences with respect to the aforementioned factors between two groups.

Apnea, sepsis, shock, and mechanical ventilation were associated with bowel perforation in NEC in the present study. Although a causal relationship cannot be simply established by this study, the relation between these factors and bowel perforation is biologically plausible. Apnea can result in hypoxia: in the hypoxic state, one of the compensatory responses which develops is intestinal vascular contraction, leading to intestinal ischemia and hypoxia, intestinal mucosal injury, and intestinal bacterial translocation and finally to NEC [16–18]. Shock can affect the circulation and aggravate the intestinal ischemia injury. Infection induces microvascular platelet-leukocyte aggregates by activating inflammatory factors, and clogged blood flow increases intestinal mucosal damage which in turn leads to intestinal necrosis and perforation. Apnea, rapid advancement of milk intake, and infection were reported as the three most significant factors for NEC [19]. Sudden onset of tachycardia and shock in preterm neonates with NEC often warns the impending perforation [20]. Mechanical ventilation was also reported as a risk factor for perforation requiring surgical intervention in infants with NEC [21]. Our study found significantly higher prevalence of apnea, sepsis, shock, and mechanical ventilation in NEC cases with bowel perforation compared to those without. Therefore, attention should be paid to these risk factors in infants with NEC. An adequate fasting time, careful monitoring of abdominal distension, and vomiting after feeding are essential to prevent bowel perforation.

NEC usually manifests as abdominal distension, vomiting, blood in stools, and feeding intolerance. Linder et al. [9] reported abdominal distension as a high-risk factor for bowel perforation in NEC. In our study, all 10 NEC cases with bowel perforation had abdominal distension, while only 74% (35/47) of the NEC cases without bowel perforation had this finding. In NEC, progression of abdominal distention should be closely monitored and, when necessary, a dynamic abdominal vertical plain radiograph may be performed. There were higher rates of pneumoperitoneum and fixed intestinal loop on abdominal radiographs in NEC group requiring surgery compared to those who did not require surgery [21]. Plain abdominal radiographs are standard imaging modalities of choice for evaluation of patients with NEC: they must be considered at any point of acute clinical deterioration. This investigation is critical because findings may be helpful for better patient management and indicate the need of surgical intervention [22].

The present study showed that NEC cases with bowel perforation were more likely to exhibit an abnormal WBC count, thrombocytopenia, significant increase of CRP, and relatively low blood pH compared to those without bowel perforation. Inflammation has been implicated as the final common pathway of NEC. WBC count and CRP are important indicators of the inflammatory response, while thrombocytopenia is a sign for severe infection.

Srinivasjois et al. [23] and Wiwanitkit [24] suggested that a significant reduction in platelet count is parallel to the progression of NEC, and, for neonates on treatment, an increasing CRP often implies the occurrence of bowel perforation. Linder et al. [9] showed a close relation between the relatively low blood pH and bowel perforation in NEC. Moreover, our finding of higher blood glucose levels in NEC cases with bowel perforation compared to those without bowel perforation agrees with previous data indicating that high blood glucose level is a risk factor for poor prognosis in NEC [9].

SNAP scoring system, including the full version consisting of 27 items of physiological indices [25] and two simplified versions (SNAP-II and SNAPPE-II) [13], is a validated tool to evaluate the health condition of neonates. The higher the SNAP scores, the worse the disease. Bonnard et al. [26] identified the utility of SNAPPE-II to guide the treatment of NEC with bowel perforation in very low birth weight (VLBW) infants. However, Ibáñez et al. [27] discouraged the application of SNAPPE-II to guide surgical intervention. In this study, we found no significant differences of SNAP-II and SNAPPE-II scores obtained upon admission between two groups. It should be noted that SNAP scores obtained at birth may indicate health condition at birth; they cannot be used to compare severity of the disease several days later.

Treatment of NEC usually includes fasting, gastrointestinal decompression, TPN, and anti-infective measures. In the current study, NEC cases with bowel perforation had a significantly longer duration of fasting and required TPN and antibiotics for longer duration compared to those without bowel perforation. This could be related to differences in the severity of NEC and longer period required for gastrointestinal functional recovery in those with bowel perforation. The higher prevalence of cholestasis and EUGR in NEC cases with bowel perforation may be explained by longer periods for infants to switch from TPN to full enteral nutrition. In a long-term follow-up of infants with and without NEC, Pike et al. [28] found significantly higher mortality and higher morbidity in intestinal and nervous system in infants with NEC than those values in the group without NEC, a fact that is similar to our findings.

The strength of our study includes the relatively complete information on demographical, clinical, and laboratory characteristics in the study population. This study has several limitations. First, the current study was based on a single center design, which may limit the generalizability of findings. Second, the sample size was relatively small, increasing the risk for type 1 and type 2 errors. Third, the treatment was given up in some infants because of financial reasons, which might have influenced the study outcomes.

In conclusion, bowel perforation in premature infants with NEC is associated with lesser gestational age, lower birth weight, apnea, mechanical ventilation, and sepsis. In addition, NEC infants with bowel perforation are at higher risk for mortality, and survivors may experience higher risk for EUGR and cholestasis. These findings highlight the need for early identification of these risk factors by close monitoring of clinical manifestations and laboratory indices in premature infants with NEC to help reduce the bowel perforation.

## Figures and Tables

**Table 1:** General characteristics in NEC infants with and without bowel perforation.

	Perforated NEC group (*n* = 10)	Nonperforated NEC group (*n* = 47)	*P*
Gestational age (weeks)^a^	31.52 ± 2.31^*∗*^	33.49 ± 2.57	0.03
Body weight (g)^a^	1550 ± 550.25^*∗*^	1967.87 ± 518.98	0.03
Male^b^	5 (50.00)	30 (63.83)	0.65
SGA^b^	3 (30.00)	7 (14.80)	0.49
Twin pregnancy^b^	5 (50.00)	13 (27.60)	0.31
Low Apgar score^b,c^	1 (10.00)	5 (10.60)	1.00
*In utero* fetal distress^b^	1 (10.00)	3 (6.30)	0.55
Cesarean section^b^	6 (60.00)	30 (63.80)	1.00
Maternal diseases^b^	4 (40.00)	17 (36.1)	1.00
SNAP-II^a^	8.38 ± 4.75	8.14 ± 6.83	0.09
SNAPPE-II^a^	12.88 ± 9.02	13.05 ± 9.96	0.96

Note: ^a^data were expressed as mean ± SD; ^b^data were expressed as the number (percentage within the group); ^c^defined as 5-minute Apgar score <7; ^*∗*^ *P* < 0.05 compared to the nonperforated NEC group.

NEC, necrotizing enterocolitis; SGA, small for gestational age; SNAP, Score for Neonatal Acute Physiology; SNAPPE-II, SNAP-Perinatal Extension-II.

Among the NEC group with perforation, 4 cases gave up treatment (all the 4 cases died according to our follow-up by telephone call), while in the NEC group without perforation, 9 cases gave up treatment due to economic reasons (6 died and 3 survived).

**Table 2:** Clinical characteristics in NEC infants with and without bowel perforation.

	Perforated NEC group (*n* = 10)	Nonperforated NEC group (*n* = 47)	*P*
Onset age (d)^a^	10.0 (7.50–22.25)	7.0 (2–14)	0.40
Time for the first enteral feeding (d)^b^	3.8 ± 3.16	5.8 ± 3.48	0.08
Speed of the increase of milk intake (cc/kg/d)^b^	4.20 ± 3.65	7.02 ± 6.48	0.07
Abdominal distension^c^	10 (100.00)	35 (74.40)	0.17
Vomiting^c^	6 (60.00)	19 (40.40)	0.43
Stool RBCs^c^	3 (30.00)	18 (38.30)	0.89
CHD^c^	4 (40.00)	9 (19.10)	0.31
Sepsis^c^	4 (40.00)^*∗*^	4 (8.50)	0.04
Apnea^c^	6 (60.00)^*∗*^	7 (14.80)	<0.01
ICH^c^	2 (20.00)	2 (4.20)	0.14
Shock^c^	3 (30.00)^*∗*^	1 (2.10)	0.02
RDS^c^	3 (30.00)	7 (14.80)	0.49
Neonatal pneumonia^c^	5 (50.00)	22 (46.80)	1.00
Blood transfusion^c^	4 (40.00)	6 (12.70)	0.12
Mechanical ventilation^c^	7 (70.00)^*∗*^	14 (29.70)	0.04

Note: ^a^data were expressed as median (interquartile range); ^b^data were expressed as mean ± SD; ^c^data were expressed as the number (percentage within the group); ^*∗*^ *P* < 0.05 compared to the nonperforated NEC group.

CHD, congenital heart disease; ICH, intracranial hemorrhage; NEC, necrotizing enterocolitis; RDS, respiratory distress syndrome.

**Table 3:** Comparison of laboratory tests in NEC infants with and without bowel perforation.

	Perforated NEC group (*n* = 10)	Nonperforated NEC group (*n* = 47)	*P*
pH value^a^	7.27 ± 0.12^*∗*^	7.37 ± 0.10	0.02
Abnormal WBC count^b,c^	6 (60.00)^*∗*^	9 (19.15)	0.02
Thrombocytopenia^b^	7 (70.00)^*∗*^	4 (8.51)	<0.01
Elevated CRP^b,d^	7 (70.00)^*∗*^	13 (27.60)	0.03
Blood glucose level (mmol/L)^a^	7.80 ± 5.40^*∗*^	3.60 ± 1.74	0.04

Note: ^a^data were expressed as mean ± SD; ^b^data were expressed as the number (percentage within the group); ^c^defined as WBC count <5.0 or >20 × 10^9^/L; ^d^defined as CRP >8 mg/L; ^*∗*^ *P* < 0.05 compared to the nonperforated NEC group.

**Table 4:** Treatments and complications in NEC infants with and without bowel perforation.

	Perforated NEC group (*n* = 4)	Nonperforated NEC group (*n* = 37)	*P*
Duration of fasting (d)^a^	17.25 ± 2.21^*∗*^	8.21 ± 1.29	<0.01
Use of carbapenem antibiotics^b^	4 (100.00)^*∗*^	6 (16.21)	<0.01
Duration of antibiotic treatment (d)^a^	21.00 ± 3.37^*∗*^	10.39 ± 3.40	<0.01
Gastrointestinal decompression^b^	4 (100.00)	17 (45.94)	0.13
Duration of TPN use (d)^a^	37.5 ± 8.10^*∗*^	22.21 ± 7.53	<0.01
Cholestasis^b^	3 (75.00)^*∗*^	5 (13.51)	<0.01
EUGR^b^	4 (100.00)^*∗*^	13 (35.10)	<0.05

Note: ^a^data were expressed as mean ± SD; ^b^data were expressed as the number (percentage within the group); ^*∗*^ *P* < 0.05 compared to the nonperforated NEC group.

## Abbreviations

CHD: Congenital heart disease
CRP: C-reactive protein
EUGR: Extrauterine growth restriction
ICH: Intracranial hemorrhage
LBW: Low birth weight
NEC: Necrotizing enterocolitis
RDS: Respiratory distress syndrome
TPN: Total parenteral nutrition
SGA: Small for gestational age
SNAP: Score for Neonatal Acute Physiology
SNAPPE-II: SNAP-Perinatal Extension-II
SD: Standard deviation
WBC: White blood cell count.
